# Comparison of Cartesian and radial acquisition on short-tau inversion recovery (STIR) sequences in breast MRI

**DOI:** 10.1590/0100-3984.2015-0197

**Published:** 2017

**Authors:** Domiziana Santucci, Sheila S. Lee, Heidi Hartman, Shyama Walgampaya, Mamdoh AlObaidy, Miguel Ramalho, Brian M. Dale, Richard C. Semelka

**Affiliations:** 1 MD, Department of Radiological Sciences, University of Rome "Sapienza", Rome, Italy, Department of Radiology, University of North Carolina at Chapel Hill, Chapel Hill, NC, USA.; 2 MD, Department of Radiology, University of North Carolina at Chapel Hill, Chapel Hill, NC, USA.; 3 PhD, Siemens Medical Solutions, Inc., Morrisville, NC, USA.

**Keywords:** Breast MRI, BLADE sequence, Cartesian sequence, Image quality

## Abstract

**Objective:**

The purpose of this study was to compare two short-tau inversion recovery (STIR) sequences, Cartesian and radial (BLADE) acquisitions, for breast magnetic resonance imaging (MRI) examinations.

**Materials and Methods:**

Ninety-six women underwent 1.5 T breast MRI exam (48 Cartesian and 48 BLADE). Qualitative analysis including image artifacts, image quality, fat-suppression, chest-wall depiction, lesion detection, lymph node depiction and overall impression were evaluated by three blinded readers. Signal to noise ratios (SNRs) were calculated. Cronbach's alpha test was used to assess inter-observer agreement. Subanalyses of image quality, chest-wall depiction and overall impression in 15 patients with implants and image quality in 31 patients with clips were correlated using Pearson test. Wilcoxon rank sum test and *t*-test were performed.

**Results:**

Motion artifacts were present in 100% and in 0% of the Cartesian and the BLADE exams, respectively. Chemical-shift artifacts were present in 8% of the Cartesian exams. Flow artifacts were more frequent on BLADE. BLADE sequence was statistically superior to Cartesian for all qualitative features (*p* < 0.05) except for fat-suppression (*p* = 0.054). In the subanalysis, BLADE was superior for implants and clips (*p* < 0.05). SNR was statistically greater for BLADE (48.35 vs. 16.17). Cronbach ranged from 0.502 to 0.813.

**Conclusion:**

BLADE appears to be superior to Cartesian acquisition of STIR imaging as measured by improved image quality, fewer artifacts, and improved chest wall and lymph node depiction.

## Abstract (pt)

**Objetivo:**

Comparar duas sequências de aquisição, cartesiana e radial (BLADE) ponderadas em *short-tau inversion recovery* (STIR), em exames de ressonância magnética de mama.

**Materiais e Métodos:**

Noventa e seis pacientes foram submetidas a exame de ressonância magnética de mama em 1,5 T (48 aquisições STIR cartesianas e 48 aquisições STIR BLADE). A análise qualitativa incluindo artefatos, qualidade de imagem, supressão de gordura, avaliação da parede torácica, detecção de lesões, linfonodos e impressão geral foi avaliada independentemente por três leitores. Os *signal to noise ratios* (SNRs) foram calculados. Foi utilizado o teste alfa de Cronbach para avaliar a concordância interobservador. Subanálises da qualidade de imagem, avaliação da parede torácica e impressão geral em 15 pacientes com implantes e qualidade de imagem em 31 pacientes com clipes cirúrgicos foram correlacionadas aplicando o teste de Pearson. Os testes de Wilcoxon *rank sum test* e Student *t* foram utilizados para comparação qualitativa e quantitativa entre as duas sequências.

**Resultados:**

Os artefatos de movimento estavam presentes em 100% e 0% dos exames de aquisição cartesiana e de BLADE, respectivamente. Os artefatos de desvio químico estavam presentes em 8% dos exames cartesianos. Artefatos de fluxo foram mais frequentes nas sequências BLADE. A sequência BLADE foi estatisticamente superior para todos os atributos qualitativos (*p* < 0,05), exceto na supressão de gordura (*p* = 0,054). O BLADE foi superior na avaliação dos implantes e clipes cirúrgicos (*p* < 0,05). O SNR foi estatisticamente superior na sequência BLADE (48,35 *versus* 16,17). Cronbach variou entre 0,502 e 0,813.

**Conclusão:**

A sequência BLADE foi superior à sequência de aquisição cartesiana de imagens na ponderação STIR, comprovada por uma melhor qualidade de imagem, menos artefatos e melhor avaliação da parede torácica e de linfonodos.

## INTRODUCTION

Magnetic resonance imaging (MRI) of the breast is a well-established technique that has substantially impacted the management of women with known or suspected breast cancer. Although dynamic gadolinium enhanced imaging has been the mainstay of breast MRI, increasingly investigators have realized the importance of a T2-weighted sequence in the protocol^(1-4)^. The American College of Radiology, in their requirements for breast MRI accreditation, lists that a "fluid sensitive" series is imperative^(5)^. Generally, most centers employ either a T2-weighted spin echo or a short tau inversion recovery (STIR) sequence, and MRI protocols include a T2-weighted unenhanced sequence with or without fat suppression^(6,7)^. Both these sequences are susceptible to motion artifact caused by a variety of factors, patient and non-patient related: patient motion during image acquisition, respiration, cardiac movements, pulsatile flow of vessels, and metallic clips or other foreign bodies such as breast implants^(8)^. Image quality and lesion detection may be compromised by these artifacts, resulting in lower signal to noise ratio (SNR)^(9)^ and decreased ability to distinguish normal and diseased structures^(10)^.

To overcome these problems, new methods for motion control and artifact reduction have been investigated^(11,12)^. Pperiodically rotated overlapping parallel lines with enhanced reconstruction (PROPELLER) acquisition technique^(13)^ has been reported as a radial *k*-space acquisition method to correct motion on T2-weighted sequences. In conventional turbo spin-echo (TSE) imaging, *k*-space is acquired in sequential parallel lines in a rectilinear pattern in the phase-encode direction, termed Cartesian acquisition.

BLADE is a version of PROPELLER, which was used for the first time in 1999^(13)^. Its use is spreading over the last years with new software developments as an alternative non-Cartesian *k*-space trajectory scheme in brain, cervical spine and head MRI studies^(9,14)^, and more recently in the abdomen^(11,15)^ and female pelvis^(16)^ exams. To our knowledge, there is only one study which reports on the application of BLADE in breast MRI^(17)^. Prior studies evaluating BLADE in non-breast applications have reported a reduction of motion artifacts and improvement in qualitative analysis of T2-weighted sequences^(8-11,13-17)^.

The aim of our study was therefore to compare various attributes of image quality between conventional Cartesian acquisition STIR and BLADE acquisition STIR in breast MRI.

## MATERIALS AND METHODS

### Patients

Two cohorts of breast MRIs were evaluated in this Institutional Review Board approved, Health Insurance Portability and Accountability Act compliant, retrospective study. We enrolled 48 consecutive breast MRI exams including BLADE sequences performed at the University of North Carolina Breast & Spine Imaging Center between August 31, 2013 and August 31, 2014 (BLADE group). We then compared it with another cohort consisting of 50 consecutive breast MRI exams including standard acquisition of Cartesian STIR (also termed turbo inversion recovery with magnitude reconstruction), acquired at our main center (University of North Carolina hospital) between March 1, 2014 and August 31, 2014 (Cartesian group).

Two examinations were excluded from the Cartesian group due to technical failure, which resulted in a final sample consisting of 96 female patients (48 from the Cartesian group and 48 from the BLADE group).

Mean age of patients was 45.7 years (range, 19-84 years) and 50.8 years (range, 20-86 years) in the Cartesian and BLADE cohort, respectively. Among these, 28 and 17 patients (58.33% and 35.42%) were in menopause, and 20 and 31 (41.67% and 64.58%) were premenopausal, for the Cartesian and BLADE cohorts, respectively.

Of the 48 Cartesian group exams, 12 patients had one or more clips and 7 patients had mono- or bilateral implants. Of the 48 BLADE group exams, 19 patients had one or more clips and 8 patients had mono- or bilateral implants.

### MRI protocol

All scans were performed at 1.5 T MR systems. The Cartesian group was performed on an Magnetom Avanto system (Siemens Healthcare; Erlangen, Germany) and the BLADE group was performed on an Magnetom Aera system (Siemens Healthcare; Erlangen, Germany). For the Cartesian group, the standard inversion-recovery sequence used Cartesian acquisition with 50% phase over-sampling, 1 mm in-plane resolution, and 3 mm slice thickness. For the BLADE group, the BLADE sequence used a spoke-wheel acquisition with no additional over sampling^(18)^, 0.8 × 0.8 mm in-plane resolution, and 4 mm slice thickness. Both sequences used TR > 4500 ms for full longitudinal recovery and both used an inversion time of 170 ms for fat suppression, but the Cartesian sequence had a slightly longer TE (73 ms vs. 65 ms). The acquisition time for both sequences was approximately 5.5 minutes (321 s for Cartesian; 327 s for BLADE).

The parameters of the different sequences were optimized to yield the best diagnostic performance for the type of sequence. The parameters are shown in Table 1.

**Table 1:** Measurement parameters for axial Cartesian and BLADE sequences.

Features	Cartesian	BLADE
TR (ms)	4700	5620
TE (ms)	73	65
Number of acquisitions	2	1
Slice thickness (mm)	3	4
Dist/gap (mm)	1	0.8
FOV (mm × mm)	350 × 320	320 × 320
Base resolution	384	384
TI for fat suppression (ms)	170	170
Phase oversampling (%)	50	No
Voxel size	1.0 × 0.9 × 3.0 mm	0.8 × 0.8 × 4.0 mm
Acquisition time (min:s)	5:35	5:45
Flow compensation	Yes	No
Phase encoding direction	R>>L	Rotating
Blade coverage (%)		82.4

TR, repetition time; TE, echo time; FOV, Field of view; TI, inversion time.

### Qualitative image analysis

One investigator, not involved in image analysis, removed all patient and scanning information from images, and presented the imaging sets on a dedicated workstation (Impax® v.6; Agfa Healthcare, Mortsel, Belgium) in a retrospective, random and independent fashion to three radiologists (readers 1, 2 and 3) who had 2, 4 and 1 years of breast MRI experience, respectively.

The radiologists were blinded to subject data, medical history and to the imaging technique. For each dataset, all readers evaluated several imaging parameters in the following order: artifacts (motion, flow, implants, clips), fat suppression homogeneity, visualization of the chest wall (sternum and pectoral muscle), lesion detection, lymph nodes depiction, image quality and overall impression for each patient. Prior to the commencement of the study, the three radiologists collectively reviewed a training dataset of 8 patients, 4 with Cartesian and 4 with BLADE, and agreed on the interpretations and scores for each parameter evaluated. This data was not included in the study.

Grading scales were used to assess the imaging findings, and all scales used for the evaluation was a modification of the Likert scale with an equal number of positive and negative statements regarding each position^(19)^. Motion, flow and chemical shift artifacts (seen as a thin band of high or low signal at fat and soft tissue boundaries) were graded on a 4-point based on the severity of artifacts (absent, mild, moderate or severe). Fat suppression was graded on a 3-point scale: non-uniform, uniform with mild suppression and uniform with strong suppression. The image quality of all evaluated images was done by means of visual assessment with regard to the presence and severity of artifacts (motion, flow, clips, implants) and homogeneity of fat suppression. Image quality was graded on a scale from 1 to 5 (1, non diagnostic; 2, poor image quality; 3, fair image quality; 4, good image quality; 5, excellent image quality). Chest wall (sternum and pectoral muscle delineation) and lymph nodes assessment used a 5-point scale: 1 indicated unacceptable depiction; 2, poor and severely blurred depiction; 3, moderate depiction; 4, clear depiction with slight blurring; 5, excellent depiction with no blurring. For lesion detection a 3-point scale was used: 1 represented non-diagnostic image; 2, possibly present or absent; 3, definitely or almost definitely present or absent. Overall image impression was graded on a 4-point scale: 1 indicated the reader was very dissatisfied; 2, somewhat dissatisfied; 3, somewhat satisfied; 4, very satisfied.

### Quantitative image analysis

For quantitative analysis one workstation was used for all measurements, in order to diminish potential variation based on equipment. One observer evaluated the images and SNRs were calculated for each exam. This investigator did not participate in image analysis. A mid-axial section was chosen and circular regions of interest (ROI) were drawn at the nipple level, at the breast center, in normal-appearing tissue of the right breast, avoiding vessels, lesions and artifacts, to obtain the signal value. A noise ROI was drawn between the two breasts in the free black air space devoid of artifacts. The SNR was calculated for each patient with the following equation: SI_breast_/SD_noise_ (signal intensity value obtained by the ROI drawn in the right breast / standard deviation of background noise)^(10)^.

### Statistical analysis

All statistical analysis was performed with the Statistical Product and Service Solutions statistical software program, version 18.0, by IBM.

Inter-observer agreement for the qualitative data was assessed with Cronbach's alpha test. Alpha value higher than 0.9 indicates an excellent internal consistency; 0.7-0.9, good agreement; 0.6-0.7, acceptable agreement; 0.5-0.6, poor agreement; and an alpha value < 0.5, unacceptable agreement^(20)^.

The 2-sided Wilcoxon rank sum test was chosen as non-parametric test to evaluate the difference between the score of the Cartesian and the BLADE sequences for all the features. Each feature was considered separately. Significance (*p*) was considered present at *p* < 0.05.

Pearson correlation test was used to compare the image quality, chest wall depiction and the overall impression scores by the three readers for the patients with implants and to compare image quality in patients with clips between Cartesian and BLADE sequences.

The 2-sided t-test was applied to the results of the quantitative evaluation (SNR values).

## RESULTS

The qualitative and agreement (alpha score) scores for each feature are reported on Table 2. Cronbach's alpha values for agreement among the three reviewers for independent qualitative data analysis ranged from 0.502 to 0.813. For the evaluation of image artifacts, chest depiction, image quality and lymph nodes depiction and overall impression the agreement was good (0.7 ≤ a < 0.9), the agreement was acceptable (0.6 ≤ a < 0.7) for fat suppression and the agreement was fair (0.5 ≤ a < 0.6) for lesion detectability.

**Table 2:** Qualitative analysis of Cartesian and BLADE sequences.

	Cartesian				BLADE			
	Reader 1^(a)^	Reader 2^(a)^	Reader 3^(a)^		Reader 1^(a)^	Reader 2^(a)^	Reader 3^(a)^	Agreement^(b)^
Image quality^(c)^	3.15 ± 0.74	2.62 ± 0.57	2.62 ± 0.82		4.00 ± 0.92	3.31 ± 0.90	3.88 ± 1.02	0.813
Fat suppression^(d)^	2.27 ± 0.76	2.29 ± 0.58	2.15 ± 0.71		2.50 ± 0.65	2.38 ± 0.57	2.38 ± 0.61	0.612
Chest depiction^(e)^	3.31 ± 0.90	2.38 ± 0.98	2.35 ± 0.93		4.15 ± 0.97	2.75 ± 1.12	3.83 ± 1.14	0.791
Lesion detectability^(f)^	2.75 ± 0.53	2.29 ± 0.71	2.00 ± 0.65		2.83 ± 0.48	2.23 ± 0.59	2.48 ± 0.62	0.502
Lymph nodes^(e)^	3.83 ± 0.95	2.92 ± 0.96	2.92 ± 1.03		3.85 ± 0.95	3.04 ± 1.13	4.10 ± 0.83	0.805
Overall impression^(g)^	2.73 ± 0.84	2.46 ± 0.77	2.23 ± 0.83		3.17 ± 1.02	2.88 ± 0.84	3.08 ± 0.77	0.762

(a) Values are expressed as mean ± standard deviation;

(b) Calculated using Cronbach’s alpha test;

(c) 1, non-diagnostic; 2, poor image quality; 3, fair image quality; 4, good image quality; 5, excellent image quality;

(d) 1, non-uniform; 2, uniform and weak; 3, uniform and strong;

(e) 1, unacceptable depiction; 2, poor and severely blurred depiction; 3, moderate depiction; 4, clear depiction with slight blurring; 5, excellent depiction with no blurring;

(f) 1, non-diagnostic image; 2, possibly present or absent; 3, definitely or almost definitely present or absent;

(g) 1, very dissatisfied; 2, somewhat dissatisfied; 3, somewhat satisfied, 4, satisfied.

**Figure 1:**
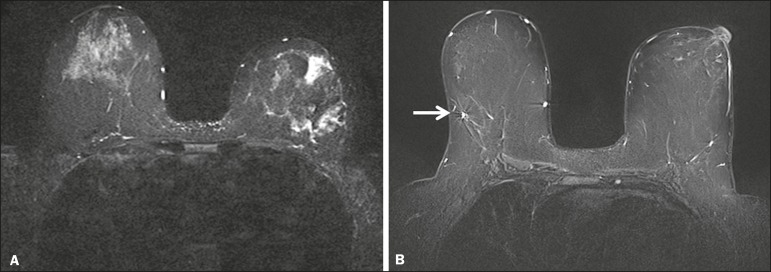
Effects of artifacts. On routine Cartesian breast images, motion artifacts are observed as ribbon-like bands extending to both sides of the thoracic wall, causing degradation of the image quality in a 46-year-old woman who had previously undergone left breast reduction (**A**). BLADE axial image in a 56-year-old woman with negative breast exam at a comparable level (**B**) show no motion artifacts. Pulsation artifacts caused by blood vessels (arrow, **B**) cause minimal degradation of portions of the BLADE image.

Motion artifacts were present in all the 48/48 (100%) in the Cartesian sequences: mild in 20 (41.67%), moderate in 19 (39.58%) and severe in 9 (18.75%). No motion artifacts were observed in the BLADE sequences (Figure 1). Regarding flow artifacts, on Cartesian sequences 17/48 patients (35.42%) showed artifacts, 14 had mild and 3 moderate; on BLADE sequences 29/48 patients (60.42%) showed flow artifacts, 14 with mild, 9 with moderate and 6 with severe artifacts. Chemical shift artifacts were present in only 4 cases, all on the Cartesian sequence.

**Figure 2:**
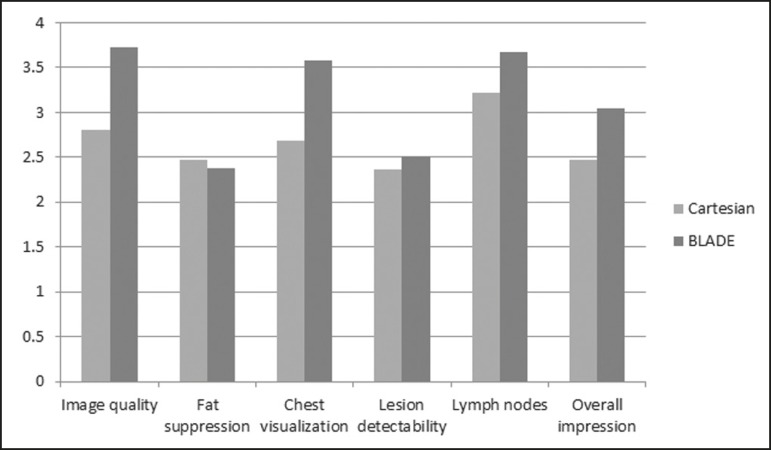
Mean values of all the readers score for each feature sorted for Cartesian and BLADE sequences.

For the Cartesian group, fat suppression was uniform (with mild or strong suppression) in 124/144 exams (86.11%); image quality was scored good or excellent in 87/144 exams (60.42%); depiction of the chest wall was good or excellent in 31/144 exams (21.53%); lesions were definitely or almost definitely present or absent in 69/144 cases (47.92%); lymph nodes depiction was good or excellent in 69/144 exams (47.92%); the overall impression of the reviewers were somewhat or completely satisfied in 59/144 exams (40.97%). For the BLADE group, fat suppression was uniform (with mild or strong suppression) in 131/144 exams (90.97%); image quality was scored good or excellent in 98/144 exams (68.06%); depiction of the chest wall was good or excellent in 83/144 exams (57.64%); lesions were definitely or almost definitely present or absent in 83/144 cases (57.64%); lymph nodes depiction was good or excellent in 89/144 exams (61.81%); the overall impression of the reviewers was somewhat or completely satisfied for 97/144 exams (67.31%). Mean value of the score assigned to each feature of Cartesian and BLADE sequences is showed in Figure 2.

**Figure 3:**
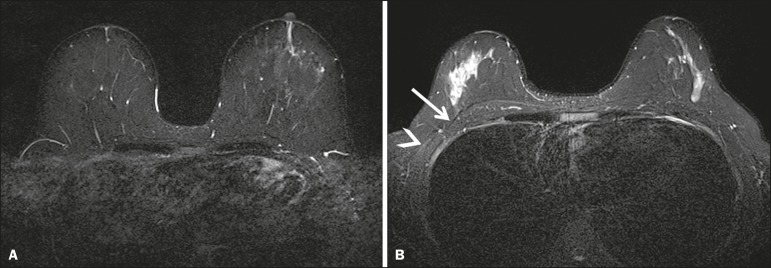
Chest wall. Heart and respiratory motion artifacts cause blurring and limit evaluation of the chest wall in routine axial Cartesian breast image of a 45-year-old woman with negative breast MRI (**A**). Fibro-glandular breast tissue, pectoralis major (arrow, **B**) and pectoralis minor (arrowhead, **B**) muscles are clearly demonstrated in axial BLADE image in a 40-year-old woman with negative breast MRI (**B**).

**Figure 4:**
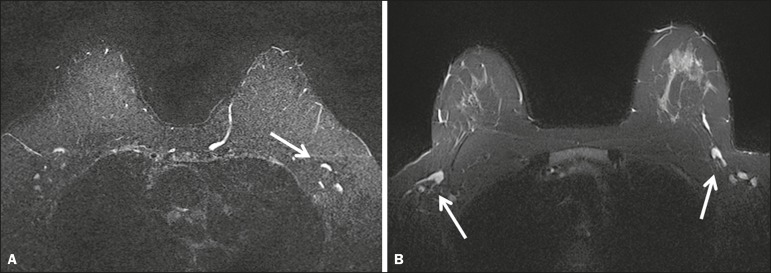
Lymph nodes. On fat suppressed images, the signal from fat in the hilum of benign lymph nodes is nulled. Axial Cartesian image in 54-year-old woman with negative breast MRI (**A**), and axial BLADE image in a 56-year-old woman with negative breast MRI (**B**). Architectural details of benign lymph nodes are not as clearly defined on the Cartesian image (arrow, **A**) compared to BLADE (arrows, **B**).

The BLADE sequence showed significantly higher image quality, chest depiction (Figure 3) and overall impression than the Cartesian sequence, with *p* < 0.0001. The BLADE sequence was also rated statistically superior to the Cartesian for lymph nodes depiction (Figure 4), with a *p* = 0.0003, and for lesion detection, with a *p* = 0.04. There was no statistical difference between Cartesian and BLADE sequences for the quality of fat suppression.

**Figure 5:**
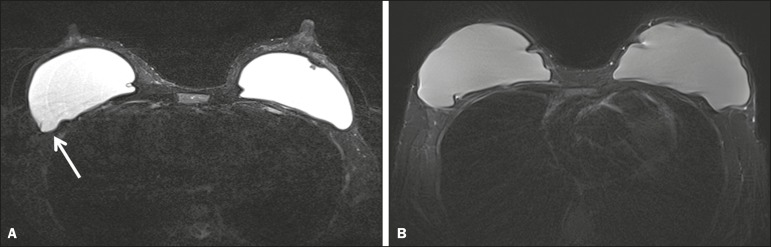
Effects of breast implants. Motion artifacts are observed adjacent to the implants and evident in the phase-encoding direction (arrow, **A**). Breast tissue close to the pectoral muscle and the pectoral muscle itself are not well assessed on the routine Cartesian breast image of a 52-year-old woman with bilateral implants (**A**). Motion artifacts are absent, leaving the breast tissue artifact-free, and the pectoral muscle and the implant contours are well delineated on BLADE axial image of a 62-year-old woman with bilateral implants (**B**).

There was a significant difference in the overall impression, chest wall depiction and in the image quality in the 15 patients with implants comparing Cartesian (8 patients) and BLADE (7 patients), with BLADE being superior, with a *p* value of 0.010, 0.002 and 0.018, respectively (Figure 5).

The image quality rating was statistically influenced by the presence of the clips: the readers' score was significantly higher in BLADE imaging than in Cartesian imaging (*p* = 0.026).

For all patients in the two cohorts, there was statistically greater SNR for BLADE compared to the Cartesian sequence with a median value of 48.35 ± 4.05 and 16.17 ± 1.20, respectively (*p* < 0.01).

## DISCUSSION

Breast MRI is an established modality for the investigation of diseases of the breast, as espoused by the American College of Radiology and the European Society of Breast Cancer Specialists, for such indications: staging before treatment planning; screening of high-risk women; evaluation of response to neoadjuvant chemotherapy; patients with breast augmentation or reconstruction; occult primary breast cancer; breast cancer recurrence; nipple discharge; characterization of equivocal findings at conventional imaging; and inflammatory breast cancer^(21,22)^. In recent years the additional value of high quality fluid sensitive sequences (T2-type) has been stressed^(3,4)^. T2-weighted sequences are employed to assess breast tissue composition and identify alterations, such as edema, inflammation, gland distortion and nipple discharge. Moreover T2 images are used to provide additional diagnostic information of the morphology and characterization of the lesions besides to evaluate diameter, shape, and invasion of adjacent structures of malignant lesions^(2)^. The results of our study have shown that a relatively new method of data acquisition, radial acquisition, possesses advantages over traditional Cartesian, as we found significant superiority of BLADE in the overall performance for breast T2-type images.

A critical aspect of maximizing information on T2-type sequences is the maximization of contrast between breast structures, which can be substantially diminished by the presence of artifacts. The most common artifacts include motion, pulsation, chemical shift and magnetic susceptibility^(23)^. Of these, motion is the most serious, and breast MR is particularly prone to, because the procedure is lengthy and somewhat uncomfortable, and the chest wall is subject to breathing artifact^(24)^.

As reported on the brain, spine, and abdomen^(9,13-15)^, we observed that image quality of the breast with a radial T2-weighted acquisition sequence was superior compared to conventional Cartesian acquisition. Our opinion is that the superiority of overall impression of BLADE primarily reflected that motion artifacts was substantially reduced, resulting in a more consistent quality high-resolution image.

Fat suppression is an essential and integral part of a breast MRI as the fat signal can mask the features of interest and interfere with the evaluation of benign lesions. The conspicuity of fibroglandular tissue and lymph nodes is greatly improved in the breast with adequate fat suppression. Our results showed that there were no differences in fat suppression between BLADE and Cartesian sequences. An important problem of the inversion-recovery imaging, obtained by a partial inversion pulse spectrally selective for fat applied intermittently throughout the pulse sequence, is sensitivity to field inhomogeneity, which may preclude satisfactory fat suppression in some cases. In our study both BLADE and Cartesian sequences where affected by heterogeneous fat suppression in almost the same percentage and this demonstrate that the *k*-space rotatory acquisition didn't improve or degrade significantly the fat suppression. It has to be recognized that different patients had different breast glandular/fat ratio, which could hamper the readers' evaluations. On the other hand, unsuppressed fat signal can also produce chemical shift artifact, which was seen only in 4 cases in Cartesian sequences.

We demonstrated that BLADE images allowed a better depiction of the chest wall, pectoral muscle and sternum. This concurs with the findings reported by Ozcam et al.^(17)^ in their study on 44 breast exams. They observed that BLADE acquisition technique provides better pectoral muscle contour assessment and better SNRs, that are essential for a good evaluation of breast exams, in particular when implants are present. Critically, in both our study and theirs, motion artifacts were almost nonexistent in BLADE acquisitions, that these authors also considered the main source for significantly improved image quality. Not only random patient motion, but also cardiac and breathing artifacts are considerably reduced comparing BLADE to Cartesian acquisition. In contrast, flow artifacts were more commonly appreciated on the BLADE compared to the Cartesian sequence. Presumably with further sequence development this can be ameliorated on BLADE, as other previous studies reported the superiority of radial acquisition in eliminating pulsation artifacts^(25)^. Despite this apparent superiority, it is possible that the BLADE technique is not yet currently in use in clinical breast MRI exams as this is a new method with few case series and due to the fact that only the newest MR units support it.

The posterior aspect of the breast is the most sensitive to motion artifact^(8,26)^. This region and the axilla often experience motion artifacts caused by patient motion, breathing, and cardiac motion, which is particularly prone to occur in axillary levels II and III^(27)^. Our results demonstrate that lymph nodes were significantly better depicted on the BLADE sequence.

The presence of breast implants has been described as a major problem for the detection of lesions close the pectoral muscle, both for their physical presence that for greater motion and flow artifacts^(26)^. Although our numbers of patients with implants were small, none-the-less we showed that the delineation of structures was much better on BLADE compared to Cartesian.

Iron-containing metal, such as surgical clips, result in signal void artifacts and can also distort the homogeneity of the magnetic field such that fat suppression may become inhomogeneous^(28)^. As clips are often placed in the breast at the site of resected cancer, this can serve to cause substantial image degradation in patients where accurate detail of potential tumor is essential. Our results showed that clip artifacts were less severe on BLADE than on Cartesian. This improvement in clip artifacts has been noted previously in the brain^(29)^.

The BLADE sequence we employed was approximately of the same duration as the Cartesian sequence. Despite the same time of acquisition, the SNR was greater with BLADE. All other factors being equal, it is always desirable to maintain a short data acquisition, and perhaps the most important reason for that is that it reduces the likelihood of patient motion. Additionally, higher SNR permits modifications such as increasing spatial resolution, which is also often an important goal^(30)^.

Our study has several limitations. The most important limitation was that the two patient cohorts were imaged on different scanners, where the Cartesian cohort was imaged on an older generation MR scanner (Avanto) compared to the BLADE sequence (Aera). This limitation was not avoidable for two primary reasons: i) the BLADE sequence was only available on the newest system; ii) all of our studies were performed on clinical patients and data interpretation was performed on retrospective analysis. All sequences were optimized for each system to maximize image quality. Moreover, the older generation system has been continuously upgraded; nevertheless, the completion of the study on different generations of devices may be considered an important bias. There were however no other selection biases of patients. As a result, our findings should be considered preliminary observations. None-the-less our findings appear sufficiently interesting that this merits further investigation with a prospective study, possibly with both sequences obtained in the same individual. Another limitation was the small number of patients with breast implants in both cohorts. Again, because of the interesting nature of our findings, this should merit a comparative study including larger numbers of patients with breast implants, possibly also with both sequences obtained in the same individual. Finally, another limitation is that our investigation was based only on general criteria such as image artifacts and image quality and did not analyze the characterization of lesions (such as margination). Nevertheless, in our preliminary results, radiologists were more certain to consider lesions as being present or absent when evaluating the BLADE sequences.

In summary, the results of our study have shown that a radial acquisition sequence, such as BLADE, results in significantly superior image quality, lesser artifacts, and improved chest wall and lymph node depiction compared to Cartesian acquisition. In particular our results indicated that BLADE sequences may be the preferred method of obtaining fluid type sequences in breast MR and they may be an excellent alternative to Cartesian turbo inversion recovery with magnitude reconstruction in patients were images are affected by motion and breath artifacts, such as old or cardiac patients. Moreover, although the low number of cases, patients with breast implants may benefit from the enhanced detail provided by BLADE acquisition and further investigation is needed.
